# Draft Genome Sequence of the Ectomycorrhizal Fungus Astraeus odoratus from Northern Thailand

**DOI:** 10.1128/MRA.00044-21

**Published:** 2021-07-01

**Authors:** Teeratas Kijpornyongpan, Weerapong Juntachai

**Affiliations:** a Department of Botany and Plant Pathology, Purdue University, West Lafayette, Indiana, USA; b Department of Biology, Faculty of Science and Technology, Chiang Mai Rajabhat University, Chiang Mai, Thailand; Vanderbilt University

## Abstract

We report the draft genome sequence of Astraeus odoratus, an edible ectomycorrhizal fungus from northern Thailand. The assembled genome has a size of 45.1 Mb and 13,403 annotated protein-coding genes. This reference genome will provide a better understanding of the biology of mushroom-forming ectomycorrhizal fungi in the family Diplocystidiaceae.

## ANNOUNCEMENT

Astraeus odoratus is an endemic ectomycorrhizal fungus (Diplocystidiaceae, Boletales, Basidiomycota) that forms a mutualistic relationship with the roots of dipterocarp trees in the northern regions of Thailand. The fungus forms fruiting bodies under the host trees from May to June (1). Despite being a popular delicacy in northern Thailand, artificial cultivation of *A. odoratus* remains unsuccessful.

To sequence the genome of *A. odoratus*, we utilized the vouchered specimen MTA3-1 collected from a community forest in Mae Tha District, Lamphun Province, Thailand. An inner tissue of the fruiting body was ground in liquid nitrogen, and DNA was extracted immediately using the GF-1 plant DNA extraction kit (Vivantis Technologies, Malaysia) using the manufacturer’s protocol. The DNA quality and quantity were determined using NanoDrop spectrophotometry and agarose gel electrophoresis. The DNA was sent to Novogene, Singapore, for high-throughput (HT) sequencing. Around 1.5 μg of DNA was used for the library preparation using the TruSeq Nano DNA high-throughput sample preparation kit (Illumina, San Diego, CA), following the manufacturer’s protocol. Sequencing was performed in paired-end (PE) mode (2 × 150 bp) on the Illumina NovaSeq 6000 platform. The 89,745,059 reads generated were assessed using FastQC 0.11.7 (2). Neither adapter sequences nor low-quality bases were detected in the reads. Ten thousand high-quality paired-end reads were randomly selected and used for BLAST searches in the NCBI nucleotide database. Of this number, 16.28%, 12.84%, and 3.10% of reads had hits to Enterobacter cloacae, Enterobacter asburiae, and Pseudomonas phage OBP, respectively. This indicates potential contaminants in our data.

The genomes of Enterobacter cloacae ATCC 13047 (GenBank accession no. KN150799), Enterobacter asburiae ATCC 35953 (BioProject accession no. PRJNA285282), and Pseudomonas phage OBP (GenBank accession no. NC_016571.1) were utilized as references for contaminant removal (3–5). The Illumina reads were mapped to these references through Bowtie 2 2.3.5.1 using --fast-local mode and -N 1 (6). Reads not mapped to the references were *de novo* assembled using ABySS 2.2.4 with the following parameters: k = 102, q = 10, and j = 8 (7). Scaffolds having a length less than 1 kb were discarded from the final assembly. The quality assessment was determined by genome statistics using QUAST 3.2 (8) and by the completeness of conserved genes using BUSCO 2.0 with the kingdom Fungi and the phylum Basidiomycota as the data sets (9). We utilized MAKER 2.31.10 for gene prediction (10). The annotation pipeline incorporated *ab initio* gene predictions from GeneMark 4.46 using fungal gene finding mode (11) and Augustus 3.2.1 using the previously trained model of Coprinopsis cinerea as an external hint (12), with homology BLAST-based prediction using the protein model of Pisolithus tinctorius (13) (GenBank accession no. PRJNA207840) as a reference. Default parameters were used for the genome assembly and annotation unless otherwise noted. Summary statistics of the assembly are provided in Table 1.

**TABLE 1:** Summary statistics for the genome assembly of *A. odoratus*

Property	Value
Assembly length (bp)	45,099,382^*a*^
No. of scaffolds	10,076
Largest scaffold (bp)	258,675
*N*_50_ (bp)	9,406
GC content (%)	49.12
Coverage (×)	107^*a*^
No. of predicted protein-coding genes	13,403
Completeness of genome assembly based on:	
BUSCO conserved gene set for the phylum Basidiomycota	1,617/1,764 (91.7%)
BUSCO conserved gene set for the kingdom Fungi	659/758 (87.0%)

a There are 89,745,059 reads from the sequencing facility. Only 67,554,725 reads were used for assembly after removing the potential contaminants.

**FIG 1:**
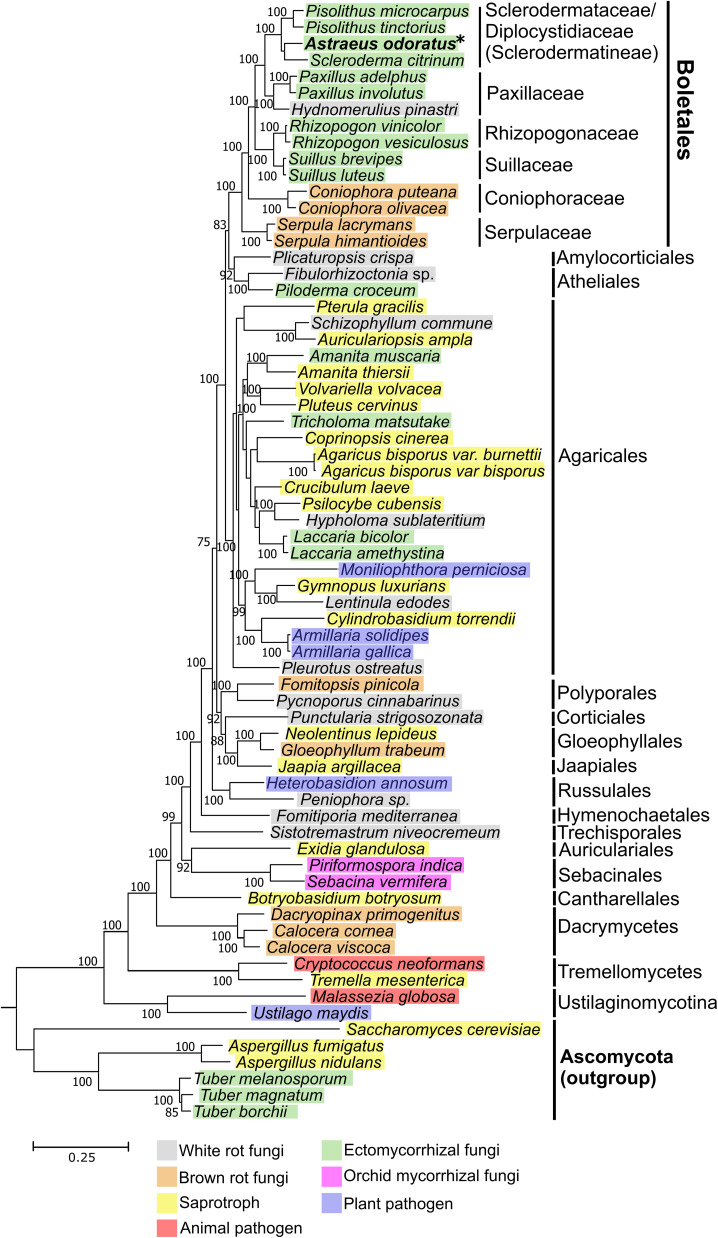
Phylogenetic placement of *Astraeus odoratus*. Sixty-eight fungal genomes were included in the phylogenomic analyses. The 124 single-copy BUSCO genes were used for phylogenomic reconstruction using the concatenation-RAxML maximum likelihood method. Only bootstrap values greater than 70 are shown as numbers next to the nodes. The taxonomic group and nutritional mode of each fungal species are indicated in the tree. The genome of *A. odoratus*, sequenced in this study, is shown in bold text with an asterisk.

We conducted phylogenomic analyses using 124 customized benchmarking universal single-copy ortholog (BUSCO) genes found in representative genomes from Basidiomycota, with some Ascomycota fungi as the outgroup (13–46). We downloaded these reference genomes from the JGI MycoCosm fungal genome portal (47). Protein sequences from each gene were aligned using MAFFT 6.903 (48); the alignments were trimmed using GBLOCKs 0.91 (49) with default parameters. Trimmed alignments of the 124 genes were concatenated and used for tree reconstruction using RAxML 8.2.9 (50) with the LG matrix with a gamma distribution for a heterogeneous rate across the sites (LGG) as a substitution model and 1,000-replicate bootstrapping. The tree (Fig. 1) confirms the phylogenetic placement of *A. odoratus* in Boletales as proposed by a previous study (51). Protein sequences, alignments, and phylogenetic trees are available in the FigShare repository (10.6084/m9.figshare.14069180).

This report represents the reference genome for the genus *Astraeus* and the family Diplocystidiaceae. This genome will be utilized to understand the biology of this ectomycorrhizal fungus, as well as how it forms fruiting bodies, which would be beneficial for industries in northern Thailand.

### Data availability.

This whole-genome shotgun project has been deposited at DDBJ/ENA/GenBank under the accession no. JADGIP000000000.1. The version described in this paper is JADGIP010000000. The sequencing reads are available under the BioProject accession no. PRJNA663954 (BioSample accession no. SAMN16189912).
